# Role of Dietary Antioxidant Supplements in Male Infertility: A Review

**DOI:** 10.7759/cureus.61951

**Published:** 2024-06-08

**Authors:** Prajakta Ghewade, Sunita Vagha, Babaji Ghewade, Pravin Gadkari

**Affiliations:** 1 Pathology, Jawaharlal Nehru Medical College, Wardha, IND; 2 Respiratory Medicine, Jawaharlal Nehru Medical College, Wardha, IND

**Keywords:** male infertility, vitamin e, vitamin c, lycopene, selenium, coenzyme q10 (coq10), dietary supplements, antioxidants

## Abstract

Infertility, which affects around 70 million couples globally, is the inability to conceive after at least a year of continuous, unprotected sexual activity. Male-related elements are involving half of all infertility cases globally. Male infertility has various characteristics, including oligospermia, asthenozoospermia, and teratozoospermia. The purpose of this study was to assess the impact of antioxidant-rich food supplements on the properties of semen, like concentration of sperm, morphology, motility, fertility rate, and damage of DNA. Terms such as coenzyme Q10, antioxidants, folic acid, vitamin C, vitamin E, male infertility, selenium and others, were used to search for relevant research papers in the PubMed database. The findings of this study demonstrated beneficial improvements in semen parameters among infertile men who consumed dietary supplements, particularly combining antioxidants like coenzyme Q10, vitamin C, and vitamin E.

## Introduction and background

The inability to conceive following at least a year of continuous, unprotected sexual activity is known as infertility [1]. Approximately 70 million couples worldwide struggle with infertility issues. The topic of male infertility is debated everywhere. According to a study, 8-12% of couples experience infertility [2]. Almost half of the world's incidences of infertility are caused by male contributors [3]. Male infertility has a number of distinguishing characteristics, including oligospermia (low concentration of sperm in semen), asthenozoospermia (complete absence of motility or reduced spermatozoa motility), and teratozoospermia (insufficient numbers of spermatozoa with proper structure) [4]. Male infertility can stem from various factors, such as smoking, radiation exposure, urinary tract infections, varicocele, poor dietary habits, and oxidative stress [5,6].

Some of the environmental factors that greatly impact male fertility are sedentary lifestyles, wearing tight clothes, using anabolic steroids, drinking excessively, emotional stress, ageing, pesticide and toxin exposure, radiofrequency electromagnetic radiation, cadmium, lead, and cytotoxic drug effects [7]. Males of reproductive age have poor dietary patterns due to the rise of unhealthy eating habits that include consuming high amounts of trans fats, sodium, and saturated fatty acids coupled with reduced intake of vegetables and fruits, which are rich sources of antioxidants and flavonoids [8,9]. Furthermore, an overabundance of foods high in trans fats and saturated fats can cause fatty acids and other dangerous lipophilic substances to build up in the environment of the testicles. This buildup may hinder spermatogenesis and lower Leydig cell testosterone synthesis [10].

Elevated oxidative stress serves as a primary driver behind conditions such as excessive increase in weight, dysbiosis of the gut, diabetes mellitus, and insulin resistance. This oxidative stress is often directly correlated with a hypercaloric diet that is unhealthy, overconsumption of trans and saturated fats, eating a diet heavy in carbohydrates and poor nutritional density [11]. The commencement of oxidative stress, a known contributor to diminished sperm quality, elevated infertility risks, and the emergence of hormonal and immunological disorders, is intricately linked to the metabolic dysfunctions and declining fertility factors previously mentioned [12]. Consequently, the presence of white adipose tissue correlates with heightened levels of pro-inflammatory cytokines, reactive oxygen species, and aromatase activity, which catalyzes testosterone's transformation into estradiol. Furthermore, hyperglycemia adversely affects sperm motility and the fertilization process [11]. ROS (reactive oxygen species) have the ability to overpower the natural antioxidant defences of the body, leading to oxidative stress [13].

The main oxidative product of sperm DNA, 8-oxodeoxyguanosine, is produced when ROS impact negatively on sperm DNA, which causes mutagenesis of DNA and fragmentation. Hence, there has been a temptation to use antioxidant supplements to alleviate the effects of reactive oxygen species on sperm [14].

## Review

Disorders of sperm production

In the testes, spermatogenesis - the process of producing sperm - takes place in structures known as seminiferous tubules. Reduced sperm production (oligospermia) or the total absence of sperm (azoospermia) can result from any disturbance in this process, including genetic defects, hormone imbalances, testicular injury, or environmental causes.

Quality and Function of Sperm

Even if sperm are created, their morphology (form) or motility (ability to move) may be incorrect, making it difficult for them to reach an egg and fertilise it. Genetics, hormone imbalances, oxidative stress, or exposure to toxins are some of the possible causes of these anomalies.

Transport of Sperm Impediment

In order to be ejaculated during sexual activity, sperm must pass from the testes through the reproductive system. Infertility can result from blockages in the ejaculatory ducts, epididymis, or vas deferens that occur during sperm ejaculation. Obstacles may arise from birth defects, medical operations, illnesses, or past injuries.

Hormonal Imbalances

The male reproductive system is largely regulated by hormones. Sperm generation, maturation, and function can be affected by hormonal imbalances in areas such as prolactin, luteinizing hormone (LH), testosterone, and follicle-stimulating hormone (FSH). Varicocele is the term for the expansion of veins in the scrotum. This condition can raise the temperature of the scrotum and interfere with the formation and quality of sperm. It is among the most prevalent and treatable reasons for male infertility.

Genetic Factors

Genetic abnormalities can impact sperm production, quality, and function. Examples of these abnormalities include Y chromosome microdeletions, chromosomal diseases (e.g., Klinefelter syndrome), and single gene mutations.

Environmental and Lifestyle Factors

Certain medications, smoking, binge drinking, drug usage, obesity, radiation or toxin exposure, and sperm production can all negatively impact a man's ability to conceive and become fertile.

Medical illnesses and Treatments

Male fertility can be affected by a number of illnesses, including diabetes, infections, autoimmune disorders, and conditions affecting the hypothalamus-pituitary-gonadal axis. In addition, procedures like radiation therapy, chemotherapy, and surgery have the potential to harm the testes and reduce the production of sperm.

For a proper diagnosis and course of therapy, it is imperative to comprehend the underlying causes of male infertility. To pinpoint the precise causes of infertility and develop individualised treatment plans, a thorough assessment conducted by a medical professional with expertise in reproductive medicine can be helpful. Numerous research studies have suggested that antioxidants, such as resveratrol, hold promise for potential applications in medicine [15]. Men experiencing infertility appear to derive significant benefits from diet and nutrition counselling. While the influence of diet is apparent in infertile patients who are either obese or underweight, many individuals with normal body weights still exhibit various nutritional abnormalities. Furthermore, optimal responsiveness to infertility treatments and fertility outcomes seems to be more influenced by dietary composition at the hypothalamus level rather than body weight alone. Furthermore, recent studies have established links between male factor infertility and inadequate intake of antioxidants, dietary deficiencies, and unhealthy eating behaviors like skipping meals or following restrictive diets [16-18].

In recent years, investigators have focused on the relation between oxidative stress and infertility in males, along with evaluating the efficacy of antioxidant supplements taken orally in improving the quality of semen among infertile men. While most studies have shown that there is a favourable relationship between antioxidants and reduction in infertility in males, conflicting findings have arisen in other studies.

The aim of this study was to assess the effects of antioxidant supplementation on parameters of semen, including morphology, concentration, DNA damage, motility, and rate of fertilization. A systematic search of papers published between 2013 and 2023 was conducted in Google Scholar and PubMed databases to gather information on the benefits of antioxidants in enhancing sperm quality. Various terms such as multivitamin, antioxidant, carnitine, vitamin E, coenzyme QQ10 (CoQ10), vitamin C, selenium, folic acid, zinc, semen, male infertility, sperm, etc. were employed during the extraction process. Only original articles, review titles and abstracts were included in the search, and no animal or laboratory testing was involved in the study. Studies examining the interaction of antioxidants with medications were excluded to focus solely on demonstrating the beneficial effects of antioxidant supplements.

Discussion

We analyzed 15 studies that evaluated the effect of supplementation of antioxidants on key characteristics of male infertility. Each study included a specific number of participants with various abnormalities such as idiopathic oligoasthenozoospermia, idiopathic infertility, varicocele, oligoasthenoteratozoospermia (OAT), etc. These participants were administered various supplements including L-carnitine, vitamin D, L-acetyl-carnitine, fructose, vitamin C, coenzyme Q10, omega-3, lycopene, selenium, folic acid, zinc, and others for durations ranging from 2.5 to 6 months. Administration of coenzyme Q10 increases seminal catalase, boosts antioxidant status, motility and sperm concentration; lycopene increases motility, sperm count and concentration; omega-3 reduces DNA fragmentation; L-carnitine increases the sperm count morphology and motility overall, elevated testosterone, inhibin levels; selenium raises sperm concentration and seminal antioxidant capacity and motility. When these antioxidants are used in combination, these actions are increased multifolds. Table 1 summarizes the findings from these 15 studies.

**Table 1:** Review of studies evaluating dietary antioxidant supplementation in cases of male infertility. DNA- Deoxyribonucleic acid, BID- bis in die/twice a day, SOD- Superoxide dismutase, CoQ- Coenzyme Q10, AA/DHA ratio- Arachidonic acid: Deoxyribonucleic acid ratio, ROS- Reactive Oxygen Species TID- ter in die/thrice a day, OAT- Oligoasthenoteratozoospermia, LH- Luteinizing hormone, FSH- Follicle stimulating hormone, SDF- Sperm DNA fragmentation, TAC-  total antioxidant capacity

Author	No. of Study Participants & Abnormality	Antioxidant supplement administered with dose	Duration	Results
Abad et al (2013) [19]	20 patients with Asthenoteratozoospermia	L- carnitine (1500 milligrams) Coenzyme Q10 (20 milligrams) Selenium (50 micrograms) Zinc (10 milligrams) Vitamin C (60 milligrams) Vitamin E (10 milligrams) Vitamin B12 (1 micrograms) Folic Acid (200 micrograms)	3 months	There was a notable improvement in DNA integrity as well as a notable rise in motility, morphology, sperm concentration and vitality.
Hadwan et al (2014) [20]	60 patients with Asthenoteratozoospermia	Zinc Sulfate (220 milligrams BID)	3 months	Boost in sperm count, semen volume and forward motility
Rawat et al (2014) [21]	83 patients with Oligoasthenoteratozoospermia	Folic acid (220 milligrams) Zinc 5 milligrams)	4 months	There were no appreciable gains in motility of sperms, morphology or concentration.
Nadjarzadeh et al (2014) [22]	47 patients with Oligoasthenoteratozoospermia	CoQ10 (200 milligrams)	3 months	An increase in seminal SOD and catalase, with a strong favourable association shown between sperm morphology and CoQ10 concentration, normal SOD and catalase.
Filipcikova et al (2015) [23]	44 infertile men	Lycopene	3 months	Notably improved seminal plasma's AA/DHA ratio
Chattopadhyay et al (2016) [24]	114 patients with idiopathic infertility	Coenzyme Q10 (200 milligrams)/day	6.5 months	Higher motility and sperm count Lower ROS level
Juan Carlos Martinez Soto et al (2016) [25]	74 infertile patients	Omega-3 (500 milligrams) TID	months	Supplementation of Omega-3 reduced DNA fragmentation and improved TAC concentrations
Gonzalez-Ravina et al (2018) [26]	60 patients with infertility	Triglycerides with 90% Omega-3	3 months	Supplementing with DHA markedly enhanced sperm motility. More dose led to more noticeable improvement right away.
Busetto et al (2018) [27]	94/Idiopathic OAT, with and without varicocele	LC (1,000 milligrams) LAC (500 milligrams) Fumarate (725 milligrams) Fructose (1,000 milligrams) Coenzyme Q10 (20 milligrams) Vitamin C (90 milligrams) Zinc (10 milligrams) Folic acid (200 micrograms) Vitamin B12 (1.5 micrograms)	6 months	A rise in the motility, concentration, and increasing motility of sperm
Nouri et al (2019) [28]	44/infertile men with oligozoospermia	Lycopene 25 milligrams /day	3 months	Lycopene supplementation increased motility, sperm count, concentration
Kopets et al (2020) [29]	83 idiopathic patients	L-carnitine/L-acetyl- Carnitine (1,990 milligrams) L-arginine (250 milligrams) Glutathione (100 milligrams) Co-enzyme Q10 (40 milligrams) Zinc (7.5 milligrams) Vitamin B9 (234 micrograms) Vitamin B12 (2 micrograms) Selenium (50 micrograms) daily	6 months	After two and four months, the percentage of treated patients with normalozoospermia was higher than that of placebo.
Hadi et al (2020) [30]	58 infertile men	L-carnitine (2 grams daily)	3 months	Increased sperm count, increased normal morphology and motility overall, elevated testosterone and inhibin levels, and decreased LH and FSH levels
Nazari et al (2021) [31]	180 infertile men with idiopathic OAT	L-Carnitine (1500 milligrams) Vitamin C (60 milligrams) Coenzyme Q10 (20 milligrams) Vitamin E (10 milligrams) Zinc (10 milligrams) Vitamin B9 (200 micrograms) Selenium (50 micrograms) Vitamin B12 (1 micrograms) Twice a day	3 months	Sperm morphology and sperm concentration improved but sperm motility did not alter.
Alahmar et al (2021) [32]	70 patients with idiopathic OAT	CoQ10 (200 milligrams /day) or selenium (200 micrograms /day)	3 months	Boost antioxidant status, motility, and sperm concentration
Alahmar et al (2023) [33]	65 patients with idiopathic OAT	Selenium 200 micrograms /day	6 months	Lowers SDF and raises concentration of sperm, seminal antioxidant capacity and motility.

Reviewing these studies, it was found that dietary antioxidant supplementation has variable effects on male infertility. When the antioxidants mentioned above were tried along with vitamin B9, B12, vitamin C, and vitamin E supplementation by Abad et al in infertile men, the results were contributory [19]. In a study done by Hadwan et al, zinc supplementation alone yielded positive effects in 60 patients with asthenoteratozoospermia which led to an increase in sperm motility, semen volume and sperm count [20].

Rawat et al in their study used zinc and folic acid supplements in 83 men with oligoasthenoteratozoospermia and there were no appreciable gains in the motility of sperms, morphology or concentration [21]. Nadjarzadeh et al (2014) performed a study on 47 patients with OAT where they supplemented 200 mg of CoQ10 for three months which resulted in an increase in seminal superoxide dismutase and catalase and there was an improvement in sperm morphology [22].

Filipcikova et al in their study included 44 infertile men who were supplemented with lycopene for 3 months and found that seminal plasma's AA/DHA ratio improved in these cases [23]. Another study done by Chattopadhyay et al in 2016 showed an increase in motility and sperm count with a lowering of reactive oxygen species level, which gives a positive effect of Co Q10 on the participating patients [24]. Supplementation of omega-3 reduced DNA fragmentation, improved total antioxidant capacity (TAC) concentrations, and enhanced sperm motility as per studies done by Juan Carlos Martinex Soto et al and Gonzalez Ravina et al respectively [25,26].

The findings of the study done by Busetto et al, where they supplemented zinc in combination with other antioxidants were similar to that done by Hadwan et al [27,20]. Nouri et al supplemented 25 milligrams/day of lycopene for 3 months in 44 infertile men with oligozoospermia and were found to increase motility, sperm count, concentration, and TAC [28]. Kopets et al found positive effects of 1990 milligrams/day of L-carnitine supplementation in 83 idiopathic patients for 6 months [29].

Another study done by Hadi et al supplemented L-carnitine 2 grams daily in 58 infertile men for 3 months and found that L-carnitine increased sperm count, normal morphology and motility overall, elevated testosterone and inhibin levels, and decreased LH and FSH levels while L-carnitine when given in combination with other supplements by Nazari et al in 180 infertile men with idiopathic OAT led to improvement in sperm morphology and sperm concentration but sperm motility did not alter [30,31].

Alahmar et al did a study where they used CoQ10 200 milligrams or selenium 200 micrograms/day in 70 patients with idiopathic OAT for 3 months and found a boost in antioxidant status, motility, and sperm concentration while when they gave selenium 200 micrograms/day in 65 patients with idiopathic OAT, it lowers sperm DNA fragmentation (SDF) and raises the concentration of sperm, seminal antioxidant capacity and motility [32,33].

## Conclusions

From the review of the aforementioned studies aiming to determine the role of dietary supplements in the treatment of infertility in males, it was observed that antioxidant supplements significantly contribute to improving treatment outcomes. These supplements have been shown to increase the concentration of sperm, motility, DNA integrity, morphology and other relevant parameters. Selenium, L-carnitine, coenzyme Q10, vitamin C, zinc, fructose, and lycopene were among the supplements frequently administered in the studies, and they demonstrated positive outcomes. Future research endeavors should focus on investigating optimal antioxidant combinations and determining appropriate dosages for administration in cases of male infertility. This would further enhance our understanding of the efficacy of antioxidant supplementation in improving male fertility outcomes.
